# A Treatise on Internal Uterine Hemorrhage

**Published:** 1835-01-01

**Authors:** 


*Traité des Hemorrhagies Internes de l'Uterus, &c.* Par A. C. BAUDELOCQUE, Docteur de la Faculté de Médicine de Paris, &c. *Ouvrage qui a remporté le Prix proposé par la Faculté de Médecine de Paris, en* 1819.—Bruxelles, 1832. 12mo. pp. 274.

*A Treatise on Internal Uterine Hemorrhage.*
By A. C. BAUDELOCQUE.

THIS work is strictly practical, consisting principally of cases, with illustrative remarks. It advances small claims to originality, since the cases are for the most part derived from the writings of others; they are, however, most judiciously chosen, and well connected by the observations of the author. The subject of abortion is necessarily involved, as a frequent consequence of internal uterine hemorrhage during gestation, and every point connected with it is elucidated by a variety of examples, with excellent practical remarks. This work is one of the best we have read on the causes and treatment of abortion.

The first chapter is perhaps the most interesting in the book, and relates to the several situations in which extravasation may occur.

I. *During the Period of Pregnancy.*

1. Blood may be effused between the uterus and epichorion, and, by detaching the ovum from its connexions, induce abortion. This is the most frequent cause of abortion in the earlier periods of gestation. In the following case this accident supervened on mechanical injury.

A woman in the third month of pregnancy received a blow on the abdomen; this was soon followed by a slight hemorrhage, which was easily arrested. Six weeks after she experienced pain in the region of the kidneys, with a disagreeable sense of weight, for which bleeding from the arm and absolute rest were prescribed. Towards the end of the fifth month labour-pains came on, and an oval black mass, of the size of a fist, was expelled, in the centre of which was found an ovum, containing the waters, and a very small fœtus in a putrid state. Seven distinct layers of blood were observed, differing in consistence, colour, and texture. (*Morlanne, Journal d'Accouchemens*, No. 12, p. 400.)

In this case the state of development of the fœtus indicates that it died at the period of the injury. The seven distinct layers of blood show that the hemorrhage took place at successive periods. The fact of the fœtus remaining in the uterus for two months, though in a decomposed state, is worthy of remark; and the case demonstrates that, when hemorrhage has occurred at an early period of pregnancy, we should not consider it certain that the child survives, until its movements become evident.

2. Between the epichorion and chorion.

CASE. The Countess ——, twenty-eight years of age, having been married six years, had a miscarriage, soon after which she again became pregnant. The day on which the menses should in ordinary course have appeared she experienced severe colic pains, which continued from seven to eight weeks, notwithstanding a state of complete repose. Frictions with ice on the lumbar and sacral regions were employed without success.

Five days after the third menstrual term, bloodletting from the arm was practised, since the pains were now attended with hemorrhage. Abortion occurred at the end of forty-eight hours.

M. Deneux examined the ovum, and found beneath the epichorion a layer of coagulated blood, of a very high colour, a line and a half thick at the sides, and five or six at the two ends. The embryo was of the size of a fly: it was thought to have died six weeks after conception. (*Journ. cité*, tome lxviii. p. 355.)

When the colic pains occurred, says M. Baudelocque, an effusion of blood must have taken place round the ovum, and at this time the fœtus ceased to live, as is proved by its state of development. The pains, occasioned at first by the menstrual turgescence, were afterwards continued by the distension caused by the effused blood, which was perhaps already extravasated at their commencement. It is difficult to conceive with what intention the frictions with ice were used; there was doubtless an error in the diagnosis.

3. Between the chorion and the amnios.

Of this no detailed cases are given; the author merely mentions that Professor Deneux has shown him several very distinct examples, and that Dr. de Kergaradec found a fibrinous concretion between the chorion and amnios, towards the insertion of the cord into the placenta.

4. Between the placenta and the uterus.

A case is related in which this took place, and appeared to be attributable to sexual intercourse during the gravid state, which M. Baudelocque considers as a frequent cause of abortion.

The following is a fatal example of a similar effusion.

A woman, thirty-six years of age, the mother of several children, was troubled, in the eighth month of pregnancy, with violent cough and fever, which lasted several days. Labour-pains supervened; and a midwife, having been in attendance for twelve hours, observed the patient fall into an alarming state of syncope. M. Delaforterie was sent for, but the patient died before his arrival. He performed the Cæsarean operation. On opening the fundus of the uterus, a pint and a half of black uncoagulated blood gushed out: this had been contained in a cavity formed between the placenta and the fundus of the uterus, the margin of the placenta having retained its natural adhesion, while the middle part was detached. The child was extracted alive, but soon died. No trace of blood, or mucus tinged with it, was found in the vagina; and the orifice of the vagina was little dilated.

It is probable that the fits of coughing had ruptured some of the vessels passing between the uterus and placenta. The pains must have been occasioned by the distension of the womb, since the state of the os uteri and vagina shows that real labour had not commenced. The child's being alive proves that the blood came from the vessels of the uterus, and not those of the placenta, and also that its effusion was recent.

5. Between the uterus, the placenta, and the external surface of the tunica decidua and chorion. This, however, is only a combination of two varieties already illustrated.

6. In the substance of the placenta.—This, M. Baudelocque observes, is of very frequent occurrence in the early periods of gestation; it also happens, however, in more advanced periods. The cases adduced in illustration are too long to be here inserted.

7. Between the umbilical vessels and the membranes which surround them.—The blood may be either in a single clot, or infiltrated.

Case. A woman, nearly at her full time, was naturally delivered of a dead child, around whose neck the cord was six times twisted. The cord was fifty-two inches in length. At the distance of a foot from the umbilicus a coagulum of blood was found, eight or ten lines long, between the membranes and the umbilical vessels: this blood had been effused from a rupture in the umbilical vein, which was varicose in several places.

This case M. Baudelocque believes to be unique: it is related as illustrating a singular cause of the death of the child. De Lamotte, Levret, and Baudelocque (senior), have recorded examples of effusion into the cavity of the amnios, in which the blood proceeded from the umbilical vessels.

7. In the cavity of the amnios.

M. Contèle gives a case in which pregnancy was mistaken for dropsy, and paracentesis was performed. This however is out of place in the consideration of uterine hemorrhage as a morbid state: bleeding from a wound may occur anywhere.

8. In the cavity of the peritoneum, which may take place at any period of gestation.

Case. A woman, who had not menstruated for six weeks, had a fall upon her knees. Some hours after colic pains commenced, and continued with violence for twenty hours, at the end of which time the menses appeared, and the pains somewhat abated. On the third day M. Littre was called: he found the patient pale, and covered with a cold clammy sweat. The abdomen was distended, the respiration difficult, the pulse small and intermittent. She complained particularly of a sense of constriction above the diaphragm, and an acute pain in the left iliac region. She soon died. A fœtus, about an inch long, was found in the abdomen, and more than four pints of blood, which had been effused from a rent in the left Fallopian tube.

II. *During Labour.*

During the process of parturition blood may be effused,

1. Between the uterus and the external surface of the placenta and membranes.

Albinus relates a case very similar to that of M. Delaforterie above cited, except that the hemorrhage occurred during actual labour; the margin of the placenta retained its adhesion, while the middle portion was separated. The event was fatal; but Albinus remarks that the patient might have been saved, had the nature of the case been understood, by rupturing the membranes, and delivering immediately. "Servare potuisset protinus rumpendo membranas infantem continentes, ut humor amnii efflueret, protrahendoque infantem cum secundinis, et si quid intus præterea concreti sanguinis remanisset. Quum enim placenta abscessit, fluit sanguis e vasis patentibus, quamdiu uterus distentus; vacuefactus autem adstringit se: sic sanguis fere conquiescit. (*Annot. Acad.* lib. i., cap. 10. p. 36.)

The practice in such a case, observes M. Baudelocque, is traced by Albinus with the hand of a master; the words *vacuefactus adstringit se: sic sanguis conquiescit*, contain the most important precept in the treatment of uterine hemorrhage, whether during gestation, labour, or the period succeeding to it.

2. In the substance of the uterus and in the abdomen.

Schmucker, in his Mélanges de Chirurgie, cites an example of a woman who became gravid six months after a Cæsarian operation, from which she had perfectly recovered. This woman was brought to bed at the full time, and almost without pain, of a dead child. Six hours after she complained of great debility, fell into a state of syncope, and died. On dissection, a mass of coagulated blood was found under the peritoneal tunic of the uterus, which distended this membrane into a kind of sac, having its parietes traversed by varicose vessels, which opened on its internal surface. A hole was found in the sac, through which blood had escaped into the abdominal cavity. The cicatrix resulting from the operation she had formerly undergone was firm and uninjured.

This fact, says M. Baudelocque, has no fellow in the history of the science. The state of the parts bears some analogy to that described in the interstitial pregnancies on which M. Breschet has written so curious a memoir. In both instances there is a cavity in the uterine parietes, dilated vessels, and an aperture communicating with the peritoneal cavity.

3. In the cavity of the amnios.—Cases of this kind are related by Levret and Baudelocque, senior, but their nature is disputed by others. For these examples we must refer to M. Baudelocque's essay.

In the second chapter the *causes* of internal uterine hemorrhage are fully and judiciously discussed. When, however, the various situations in which extravasation may take place are ascertained, the connexions of the blood-vessels duly considered, and the local and constitutional effects of the gravid state properly understood, the causes of hemorrhage may be arrived at by easy deductions; we shall therefore content ourselves with recommending this chapter to the attention of the reader, and pass on to the third, which treats of the

### *Symptoms of Internal Uterine Hæmorrhage.*

These differ in the earlier and latter periods of pregnancy. In the earlier periods the accident is frequently preceded by restlessness, dull pains in the pelvis, sense of weight about the rectum, and ardor urinæ. Pain in the region of the kidneys, severe griping and feeling of tension in the hypogastrium, soon succeed. The occurrence of the hemorrhage is often immediately preceded by a great increase of pain; sometimes the exact time of its commencement cannot be ascertained. In all the cases on record, the colic and renal pains, and the sense of weight about the rectum, have been observed to continue obstinately till the moment of abortion. The precursory symptoms are those of sanguineous congestion in the uterus; it is only the *obstinate continuance* of colic and renal pains that is diagnostic of the effusion of blood: where such pains arise merely from congestion they may be overcome.

General symptoms sometimes accompany the local, such as lassitude, headach, slight shiverings; the pulse is sometimes strong and hard, at others small, but incompressible; the countenance is animated, the eyes sparkling, and the general heat increased; the respiration is sometimes embarrassed, and the symptoms altogether are those of febrile excitement. In some cases the constitutional symptoms present themselves first: a state of general plethora is followed by congestion in the uterus, which terminates in the rupture of vessels, and internal or external hemorrhage. All these symptoms are most liable to occur in women who menstruate abundantly, and are subject to colic pains at the menstrual periods; such symptoms also occur more frequently at these periods than at any other.

Occasionally the symptoms of internal hemorrhage are preceded, accompanied, or followed, by external discharge of blood. In the early periods of pregnancy blood may be accumulated in the uterus, and cause the death of the child, without any symptoms which lead to a suspicion of the mischief.

At a more advanced period of gestation the extravasation of blood always causes dull and deep-seated colic pains, pain in the region of the kidneys, and tension and weight in the hypogastrium. Frequently the movements of the child, after having been very strong, cease to be perceptible, while the volume of the uterus increases, and it becomes harder and less compressible. When the loss of blood is inconsiderable, no other symptoms than those above mentioned present themselves; but, if it increases, the woman gradually loses her strength, and becomes pale; the pulse grows feeble, and the abdomen is greatly enlarged. Tinnitus aurium, and flashes of light before the eyes, with convulsions and syncope, soon terminate in death, if the patient be not relieved by the resources of art. External discharge of blood may be superadded to these symptoms. There is great variety in the duration of the symptoms of internal hemorrhage: sometimes they are speedily terminated by abortion, at others they disappear entirely, and gestation continues; or the symptoms may recur at the end of one or two months, and finally occasion miscarriage. Most frequently, however, the renal pains and sense of weight about the rectum continue till the occurrence of abortion, which takes place at the end of eight, ten, or fifteen days, a month, two months, or more. At an early period all the symptoms are occasioned by the presence of the effused blood, for the loss is not in itself sufficient to affect the constitution. At a later period the mere presence of the blood is of small moment; the bad symptoms arise from the effects of loss of blood on the mother and child.

When internal hemorrhage occurs during labour, certain symptoms are observable, in addition to those above enumerated. Levret has remarked, that, in the intervals of the pains, the volume of the uterus progressively increases; and Leroux has detected an obscure feeling of fluctuation. There is frequently external discharge of blood; and, when the cessation of a pain allows the head to recede, clots of blood are discharged, in larger or smaller quantity. The pains also, which are always slow and feeble in such cases, become less frequent as the extravasation increases, and at last usually cease altogether; contrary to the erroneous statements of some authors, who assert that they become more violent and frequent.

The symptoms of internal hemorrhage after the expulsion of the child, though they have become familiar to practitioners only of late years, are now sufficiently so to render it unnecessary to dwell on this part of the subject; the remarks of M. Baudelocque may, nevertheless, be perused with advantage.

The third section of this chapter is devoted to diagnosis. The diagnosis of every disease springs from an accurate knowledge of its symptoms: these having been sufficiently detailed with reference to most of the varieties of internal hemorrhage, we shall confine ourselves to M. Baudelocque's observations on the diagnosis of *utero-peritoneal hemorrhage*, which is attended with more difficulty than that of most other kinds.

During pregnancy, the only indications of this accident are pain, and the ordinary symptoms attendant on large loss of blood. Violent pain in the abdomen suddenly supervenes, which is generally confined to a particular spot. The pain soon extends, and is accompanied with prostration of strength, fainting, paleness of the countenance, intermittent, and sometimes extremely rapid pulse, vomiting, and coldness of the surface; these are speedily followed by death, previously to which the pain generally ceases. The intellectual functions are usually unimpaired.

It sometimes happens, when the extravasation proceeds slowly, that the inflammation has time to extend over the peritoneum; and the symptoms of inflammation, united with those arising from loss of blood, render the diagnosis exceedingly obscure. The following case, which occurred to M. Dance, is recorded in the memoir of M. Breschet.

—— Fouchaux, a servant, thirty-four years of age, of fine stature, well formed, and of a sanguineo-nervous temperament, was admitted into the Hôtel Dieu, on the 21st July, 1825. M. Dance was then a house-pupil of this institution. This gentleman thought, from the appearance of the patient, that she was labouring under acute peritonitis. After much questioning, the following particulars were elicited from her. She had been married at the age of eighteen, and had borne three children by the time she was twenty-four, one of which only survived; the third died in the birth. She had always enjoyed good health, and all her labours had been easy. For about three months the menses had been suppressed, but she felt convinced that this circumstance was unconnected with pregnancy. She had experienced a feeling of weight in the region of the kidneys, and uneasy sensations in the hypogastrium, at each period when the menses ought to have appeared, but her general health had not suffered. On the 20th of July she rose in the morning, without any feeling of indisposition, and went about her usual avocations. At one o'clock P.M. she was sent on an errand to the Marché St. Jacques, and on her way home was suddenly seized with a violent pain around the navel, resembling colic, succeeded by syncope, which continued for an hour. She was carried to a neighbouring house, where she came to herself, but remained pale and exhausted, and the abdomen became more and more painful. During the night she suffered great anguish, and was very restless; she slept, however, at intervals. Vomiting occurred, but soon ceased, and did not return. The bowels were not opened, and the urinary secretion was suppressed. A small quantity of blood was discharged *per vaginam*. A physician who was sent for prescribed nothing but a quieting draught.

M. Dance first saw her about two in the afternoon of the 21st July. At that time her face was pale, and her physiognomy indicated abdominal disease. The eyes were dull and sunk, the lips livid, the voice feeble, the intellectual functions not at all impaired. The belly was tumid, hard at its inferior part, and tender over its whole surface, but particularly in the iliac regions. The limbs were cold, the belly of natural temperature; the pulse very small, and threadlike; the tongue pale, and whitish in the centre. Much thirst; no vomiting.

M. Dance was naturally puzzled with these symptoms. The tumefaction and tenderness of the abdomen indicated acute peritonitis: but whence the immediate accession of such alarming symptoms in a young person previously in good health? Had there been a sudden perforation of the stomach or intestines, with effusion of their contents, and secondary peritonitis? The patient had shown no symptoms of disease in the alimentary canal. Was the case one of abortion, which the patient wished to conceal? The absence of the menses for three months induced a suspicion that this was the case, notwithstanding the woman's declaration that she was not pregnant; and M. Dance thought it necessary to examine. He found the body of the uterus harder and heavier than natural; the cervix was shortened, and softened at its anterior extremity; the os uteri was circular, and would admit the point of the finger. M. Dance was quite convinced that she was pregnant. He observed that there was no blood on the end of the finger when it was withdrawn. He intended to empty the bladder, with a view of examining the uterus more accurately: he deferred this, however, till his evening visit, and prescribed leeches and emollient fomentations. He had scarcely left the room when the patient expired.

On examining the body, twenty-four hours after death, numerous clots of blood were found in the abdomen, and especially in the cavity of the pelvis: about five or six pounds appeared to have been effused. Blood was also infiltrated between the layers of the mesentery, and in the subperitoneal cellular substance. The author adds, that he omits, as foreign to the illustration of his subject, the details of an interstitial conception, which was present in this case.

The fourth chapter treats of the *state of the effused blood*, as to consistence, colour, &c., and also of the *prognosis of internal hemorrhage*. The prognosis in cases of this kind will generally be sufficiently obvious, when the nature of the case is distinctly understood; we proceed therefore to the fifth and last chapter, on the *treatment*.

When hemorrhage to any considerable extent has actually occurred, the practice to be followed is pretty well agreed on among well-informed obstetricians; the prophylactic treatment, however, though not less important, is less obvious; we shall therefore condense some of the author's observations on this point.

It has been already stated that internal hemorrhage during pregnancy occurs most frequently in women who have the menses abundant, and are subject to colic pains at the period of their occurrence: the precepts about to be laid down ought therefore to be particularly observed with reference to such patients.

All the causes of internal hemorrhage may be resolved into those which violently detach the placenta from the uterus, or those inducing local or general plethora, which, by over distending the uterine vessels, occasions a rupture of them.

Pregnant women should avoid exposure to inclement weather and extreme vicissitudes of temperature, which, by exciting pulmonary catarrh and cough, become a source of great danger; and, if violent cough supervene during gestation, recourse cannot be had too early to the means of removing it.

The clothing of pregnant women should neither be too light nor too heavy; all tight pressure should be avoided. Mauriceau, Delamotte, and Baudelocque, sen., have recorded examples of uterine hemorrhage resulting from compression of the abdomen; and Madame Boivin relates the case of a woman who miscarried twice in succession, from wearing leather stockings for varicose veins in the legs. Stays should be discarded. Soft beds, and too warm bedclothes, are frequent causes of abortion, and should therefore be avoided. Warm baths are always hurtful; and Tymoni, a physician of Constantinople, assures us that the Turkish women, who are addicted to their excessive use, are very subject to all kinds of hemorrhage.

Tepid baths are useful as a means of cleanliness, but they should be avoided at the times corresponding to the menstrual periods. Cold bathing may be useful or dangerous, according to circumstances. It is injurious to women of a plethoric habit, and who menstruate abundantly; it is serviceable to women of a feeble constitution and lymphatic temperament, and those whose uterine system is inactive. A pregnant woman, however, should never use a cold bath from the first, but begin with a tepid one, and have its temperature reduced by degrees. Warm hip baths and pediluvia are to be avoided. The diet of a pregnant woman should be moderate; errors in quantity do more harm than those in quality; heating and highly-spiced dishes, however, strong wines, alcoholic fluids, and coffee, are objectionable. It is of great importance that the bowels be kept sufficiently open, and the urine regularly evacuated. Gentle purgatives may be used, if necessary, but must be cautiously avoided at the menstrual periods, or when there are any symptoms of uterine congestion. Emetics should never be had recourse to, unless for some urgent reason, since they may excite abortion. A case of this kind is cited by our author from Smellie. Moderate exercise on foot is advisable, but all kinds of exercise attended with jolting are injurious. The bad effects of sexual intercourse and moral excitements are well known.

It sometimes happens that internal uterine hemorrhage occurs without apparent cause, or supervenes immediately upon the cause. Art in these cases is of little avail; but when symptoms of uterine congestion merely present themselves, with or without apparent cause, the prompt intervention of art may prevent further evil from ensuing. Mauriceau relates a case, in which uterine hemorrhage occurred in a woman six weeks gone with child, and continued, with rare intermissions of a few days, for three months. Nevertheless, by means of a constant recumbent position, abstinence from sexual intercourse, and two venesections, the patient reached her full time, and was delivered of a large and healthy child. (*Obs.* 612, p. 502.) Other cases are related from the same author, and the following one from Delamotte.

A large window-shutter fell on the abdomen of a woman three months gone with child, and occasioned violent pain. A slight hemorrhage occurred immediately after. This patient found, contrary to ordinary observation, that she lost more blood when in the recumbent posture than when sitting or standing. Delamotte wisely preferred the fact to the rule; he desired her to avoid the recumbent posture, but to keep perfectly still, and twice ordered her to be bled from the arm. The bad symptoms disappeared, and Delamotte heard no more of his patient till she had reached her full time, when he delivered her of a healthy boy.

M. Baudelocque observes, that bloodletting is particularly useful in internal uterine hemorrhages occasioned by violence, and that, when employed with judgment and caution, it may often be useful, and is never injurious. The following is a case illustrative of its application.

Madame C—, æt. twenty-one, of a nervous and lymphatic constitution, was accustomed to menstruate during five days, and to be afflicted during that time with colic and renal pains. She had two miscarriages in the first year after her marriage, in both instances at the twelfth menstrual period. She became a third time pregnant three months after the second abortion. Conception took place between the 17th and 20th November, 1814, immediately after the flow of the menses. She instantly abstained from carriage-exercise, sexual intercourse, and stimulating food and drinks. On the 9th December she looked paler than usual, the eyes were hollow, and the pulse feeble; there was slight pain in the region of the kidneys, and tension of the hypogastrium. These symptoms continued for five or six days, and gradually disappeared. She kept constantly in her chamber, and was often on the sofa, till the 7th January, when she experienced some pain in the kidneys. On the 8th, the whole surface of the body was discoloured, and the roots of the nails of a violet tint; the eyes were sunk, the hands and feet cold, the hypogastrium tumid and tense, and the pulse so small as to be hardly perceptible; at intervals there were colic pains. M. Deneux ordered four ounces of blood to be drawn from the arm. The pulse immediately rose; the temperature became more equal; the pains sensibly diminished in the course of the day; and, together with the swelling of the hypogastrium, ceased entirely on the morrow. The patient continued the use of the sofa, and abstinence from sexual intercourse.

The same symptoms reappeared on the 5th February. Bleeding was prescribed, but deferred, because the patient was thought by her friends to be very weak.

On the morning of the 7th, a sense of weight about the rectum, painful micturition, pain in the hypogastric region, and oozing of blood from the vagina, were superadded to the former symptoms. The colic and renal pains were stronger and more frequent; the os uteri was tumid and painful when touched, and the parietes of the vagina were swelled, tender, and extremely hot.

M. Deneux drew a small quantity of blood (*une palette*), and prescribed complete rest, chicken-tea, infusion of linden-flowers, and emollient enemata. These measures were eminently successful; she passed a good night, and the next day was almost as well as usual. She confined herself to bed or the sofa till the 18th.

The symptoms recurred in a minor degree on the 3d March and 2d April, and were again removed by venesection. Finally, blood was again drawn on the 1st July, (this being nearly the eighth menstrual period,) for a congestion of blood in the lungs. She was safely delivered at the full time.

Madame C— has since borne two children, without any necessity for bleeding during gestation.

Too much blood should not be drawn at one venesection.

The author has observed many instances in which the too copious abstraction of blood was fatal to the child. If syncope be induced this result sometimes follows; and it is hence prudent to bleed pregnant women in a recumbent posture, in order to diminish the probability of fainting.

Ranchin, Rivière, Mauriceau, Pechlin, &c., advise that the blood should be abstracted gradually, the finger being applied at intervals over the orifice in the vein. Bleeding from the arm is to be preferred to the application of leeches in cases of uterine congestion, since the latter practice conjoins a revulsive with an evacuant effect, and determines a flow of blood to the uterine system.

The prophylactic measures against hemorrhage during labour are widely different from those above mentioned. The feeble and incontractile state of the uterus, which gives rise to this accident, is to be obviated by a generous diet during pregnancy, by the use of chalybeate tonics, by exercise, the cold bath, and even seabathing, when not otherwise contraindicated. The author has found great advantage from the exhibition of the *secale cornutum*, for the prevention of hemorrhage after the expulsion of the child. In many cases where he has administered it, he has found the lochia more scanty than usual, and their appearance delayed till several days after delivery. When there is any reason to apprehend internal hemorrhage, it is advisable to administer a small dose, as ten or twelve grains, immediately after the expulsion of the placenta. The increased hardness of the uterine tumor speedily affords evidence of the action of the remedy.

The *curative treatment* of internal hemorrhage occupies the remainder of the volume: on this, however, we shall not enter, because we have already exceeded our limits, and the subject is one very familiar to all well-informed practitioners. The reader will find some excellent observations on the use of the secale cornutum, and on the practice of plugging the vagina. This work is highly creditable to the industry and judgment of the author. Every important precept is amply illustrated by examples derived from his own observation, or the works of the best practical writers. The practitioner will find this a very valuable book of reference.

